# Patterns of antimicrobial resistance in *Salmonella* isolates from fattening pigs in Spain

**DOI:** 10.1186/s12917-022-03377-3

**Published:** 2022-09-03

**Authors:** Kendy Tzu-yun Teng, Marc Aerts, Stijn Jaspers, Maria Ugarte-Ruiz, Miguel A. Moreno, Jose Luis Saez, Soledad Collado, Cristina de Frutos, Lucas Dominguez, Julio Alvarez

**Affiliations:** 1 VISAVET Health Surveillance Centre, Universidad Complutense, Madrid, Spain; 2 Department of Veterinary Medicine, College of Veterinary Medicine, National Chung Hsing University, Taichung City, Taiwan; 3 Interuniversity Institute for Biostatistics and Statistical Bioinformatics, Hasselt University, Diepenbeek, Belgium; 4 Data Science Institute, Hasselt University, Diepenbeek, Belgium; 5 Department of Animal Health, Faculty of Veterinary Medicine, Universidad Complutense, Madrid, Spain; 6 Subdirección General de Sanidad e Higiene Animal y Trazabilidad, Dirección General de La Producción Agraria, Ministerio de Agricultura, Pesca y Alimentación, Madrid, Spain; 7 Laboratorio Central de Veterinaria (LCV Algete), Ministerio de Agricultura, Pesca y Alimentación, Madrid, Spain

**Keywords:** Multidrug resistance, Multivariate analysis, Typhimurium, 1,4,[5],12:i:-, Bayesian network analysis, Hierarchical clustering

## Abstract

**Background:**

Swine are considered a major source of foodborne salmonellosis, a public health issue further complicated by the circulation of multidrug-resistant *Salmonella* strains that threaten the safety of the food chain. The current study aimed to identify patterns that can help to understand the epidemiology of antimicrobial resistance (AMR) in *Salmonella* in pigs in Spain through the application of several multivariate statistical methods to data from the AMR national surveillance programs from 2001 to 2017.

**Results:**

A total of 1,318 pig *Salmonella* isolates belonging to 63 different serotypes were isolated and their AMR profiles were determined. Tetracycline resistance across provinces in Spain was the highest among all antimicrobials and ranged from 66.7% to 95.8%, followed by sulfamethoxazole resistance (range: 42.5% − 77.8%), streptomycin resistance (range: 45.7% − 76.7%), ampicillin resistance (range: 24.3% − 66.7%, with a lower percentage of resistance in the South-East of Spain), and chloramphenicol resistance (range: 8.5% − 41.1%). A significant increase in the percentage of resistant isolates to chloramphenicol, sulfamethoxazole, ampicillin and trimethoprim from 2013 to 2017 was observed. Bayesian network analysis showed the existence of dependencies between resistance to antimicrobials of the same but also different families, with chloramphenicol and sulfamethoxazole in the centre of the networks. In the networks, the conditional probability for an isolate susceptible to ciprofloxacin that was also susceptible to nalidixic acid was 0.999 but for an isolate resistant to ciprofloxacin that was also resistant to nalidixic acid was only 0.779. An isolate susceptible to florfenicol would be expected to be susceptible to chloramphenicol, whereas an isolate resistant to chloramphenicol had a conditional probability of being resistant to florfenicol at only 0.221. Hierarchical clustering further demonstrated the linkage between certain resistances (and serotypes). For example, a higher likelihood of multidrug-resistance in isolates belonging to 1,4,[5],12:i:- serotype was found, and in the cluster where all isolates were resistant to tetracycline, chloramphenicol and florfenicol, 86.9% (n = 53) of the isolates were Typhimurium.

**Conclusion:**

Our study demonstrated the power of multivariate statistical methods in discovering trends and patterns of AMR and found the existence of serotype-specific AMR patterns for serotypes of public health concern in *Salmonella* isolates in pigs in Spain.

**Supplementary Information:**

The online version contains supplementary material available at 10.1186/s12917-022-03377-3.

## Introduction

The emergence of antimicrobial resistance (AMR) causes a substantial burden on human health and has a strong impact on the economy. Up to 33,000 deaths are attributed or related to multidrug-resistant (MDR, usually defined as resistance to 3 or more antimicrobial classes [1]) bacterial infections every year in Europe, costing 1.5 billion Euros [2]. This phenomenon is well-characterized in the case of non‐Typhoidal *Salmonella* [3, 4], one of the main foodborne zoonoses worldwide [5], for which resistant isolates have been associated with more severe conditions and higher mortality [6, 7].

Resistant non‐Typhoidal *Salmonella *strains that show multidrug-resistant phenotypes, such as Typhimurium phage type DT104 (characterized by penta-resistance to ampicillin, chloramphenicol, streptomycin, sulfonamides and tetracycline ─ ACSSuT), are of special concern from a public health perspective [8]. Many phenotypic resistant patterns (resistotypes) have been reported in* Salmonella*, with their distribution and prevalence changing over time and space [9–11]. The interplay between *Salmonella* serotypes and resistotypes further complicates the picture, since the serotype of *Salmonella* has been shown to be associated with the pathogenicity, virulence, host range, and, most relevant here, AMR profile [12–14], and certain serotypes tend to show higher levels of resistance. For example, the proportion of MDR strains among Derby human clinical isolates in Europe in 2017 was lower than among Typhimurium and 1,4,[5],12:i:- [12]. Liao et al., (2019) also suggested serotype-specific evolutionary patterns of AMR in Typhimurium, Dublin, and Newport [14].

In Spain, approximately 35% of foodborne salmonellosis cases have been attributed to pigs [15], and a high proportion of non‐Typhoidal *Salmonella* isolates recovered from fattening pig carcases and caecal contents showed a multidrug-resistant phenotype [12]. To enable effective control programmes aiming at reducing multidrug resistance in swine-originated *Salmonella*, it is essential to understand factors related to the presence of resistance and co-resistance. However, there has been limited research on the distribution of AMR profiles of swine *Salmonella* in Spain. One study, using faecal samples from fattening pigs across Spain in 2003, reported the resistoypes of 290* Salmonella* isolates to 17 antimicrobials, with a high percentage of MDR Typhimurium, including ACSSuT Typhimurium, and 4,5,12:i:-[9].

Here, we aimed to provide information about the phenotypic AMR profiles found in Salmonella isolates recovered from pigs in Spain from 2001 to 2017 through a nationwide sampling system. We investigated (a) the temporal and spatial trends of resistance to individual antimicrobials, (b) relationships between resistance to different antimicrobials and (c) resistotypes, their associations with common serotypes in pigs in Spain and their spatial distribution. The epidemiological evidence generated by the current study is expected to help to identify important relationships between resistance to specific antimicrobials and to shed some light on the underlying mechanisms of the presentation of resistotypes that could help to design preventive and mitigation measures.

## Methods

### Study population and sample collection

The VISAVET Health Surveillance Centre in Madrid has been involved in the national antimicrobial resistance surveillance for *Salmonella* detection in food-producing pigs, commissioned by the Spanish Ministry of Agriculture, Food and Fisheries, during the study period. Isolates were recovered from faecal samples from fattening pigs collected at abattoirs of high slaughter capacity (7–20 abattoirs located in ≥ 50% of the provinces in Spain each year) and represented independent epidemiological units (i.e., farms). More details on the sampling strategy can be found in Teng et al., 2020 [16].

Information about *Salmonella* isolates that were collected between 2001 and 2017 and underwent antimicrobial susceptibility testing (AST) such as the date of sampling, the province and the autonomous community of the originated farm of the sampled pigs, the serotype, and AST results were included in the current study. Bacteriology methods can be found in Teng et al., 2020 [16]. Briefly, isolation of *Salmonella* was performed according to ISO 6579:2002/Amd 1:2007 before 2017 and ISO 6579–1:2017 for 2017 samples. Serological typing was conducted based on the White-Kauffmann-Le Minor scheme [17]. Minimum inhibitory concentrations (MICs) for various antimicrobials for isolates were determined using the two-fold broth microdilution reference method, according to ISO 20776–1:2006. For each of the tested antimicrobials, a binary outcome was constructed based on the EUCAST epidemiological cut-off value (ECOFF) as suggested by The European Committee on Antimicrobial Susceptibility Testing (EUCAST; accessed on 2020 Mar 17) [18]. More specifically, resistant (i.e., non-wild-type) isolates have a MIC larger than the ECOFF, while susceptible (i.e., wild-type) isolates have a MIC smaller than or equal to the ECOFF. The ECOFFs, as well as the ranges for each antimicrobial and year, are now provided in Supplementary File 1. Although the ranges varied slightly across the study period, this did not affect the interpretation since the ECOFFs were always within the tested ranges.

### Statistical analyses

#### Data process

Data cleaning, management and analyses were conducted in Microsoft Excel 2013 (Microsoft Corp.) and R programme version 3.5.2 (R Core Team) in RStudio interface version 1.2.1330 (RStudio Team) [19, 20].

Eleven antimicrobials for which AST results were available during most of the study period were considered in the analysis. Because AST was conducted for more antimicrobials in 2008 − 2017, the data were grouped into two datasets: one with more years and fewer antimicrobials being tested, and the other with more antimicrobials being tested and fewer years (Table 1). The first dataset (D_am7) included the AST results for seven antimicrobials [tetracycline (TET), chloramphenicol (CHL), ciprofloxacin (CIP), nalidixic acid (NAL), gentamicin (GEN), florfenicol (FFC), and cefotaxime (CTX)] from 2001 to 2013. The second dataset (D_am10) contained the AST results for the aforementioned antimicrobials except for FFC, as well as sulfamethoxazole (SMX), ampicillin (AMP), trimethoprim (TMP) and ceftazidime (CAZ), which were tested in isolates collected from 2008 to 2013 and 2017 (no sampling was conducted in swine in 2014 and 2016, and no results were available for 2015). The year isolates were recovered and their serotype was also included in the datasets. D_am7 and D_am10 datasets were analysed separately except in the analyses for spatial trends, which were conducted for individual antimicrobials using all data across the years [and including an additional antimicrobial, streptomycin (STR)]. If not otherwise specified, isolates with missing values on the AST results were removed from the analyses.

**Table 1:** Number of *Salmonella* isolates from pigs collected through the Spanish Veterinary Antimicrobial Resistance Surveillance Network programme that were tested for susceptibility to each of the eleven antimicrobials from 2001 to 2017

**year**	**TET** ^a,b^		**CHL** ^a,b^		**CIP** ^a,b^		**NAL** ^a,b^		**GEN** ^a,b^		**FFC** ^a^		**CTX** ^a,b^		**SMX** ^b^		**AMP** ^b^		**TMP** ^b^		**CAZ** ^b^		**Total count**
	**R** ^c^ **(%)**	**S** ^d^	**R (%)**	**S**	**R (%)**	**S**	**R (%)**	**S**	**R (%)**	**S**	**R (%)**	**S**	**R (%)**	**S**	**R (%)**	**S**	**R (%)**	**S**	**R (%)**	**S**	**R (%)**	**S**	
2001	69 (94.5%)	4	26 (35.6%)	47	13 (17.8%)	60	11 (15.1%)	62	14 (19.2%)	59	5 (6.8%)	68	2 (2.7%)	71	-	0	-	0	-	0	-	0	73
2002	44 (97.8%)	1	15 (33.3%)	30	4 (8.9%)	41	3 (6.7%)	42	2 (4.4%)	43	3 (6.7%)	42	2 (4.4%)	43	-	0	-	0	-	0	-	0	45
2003	72 (84.7%)	13	26 (30.6%)	59	4 (4.7%)	81	3 (3.5%)	82	9 (10.6%)	76	2 (2.4%)	83	5 (5.9%)	80	-	0	-	0	-	0	-	0	85
2004	99 (81.8%)	22	34 (28.1%)	87	9 (7.4%)	112	7 (5.8%)	114	7 (5.8%)	114	10 (8.3%)	111	0 (0%)	121	-	0	-	0	-	0	-	0	121
2005	94 (73.4%)	34	21 (16.4%)	107	10 (7.8%)	118	8 (6.2%)	120	10 (7.8%)	118	2 (1.6%)	126	2 (1.6%)	126	-	0	-	0	-	0	-	0	128
2006	96 (92.3%)	8	33 (31.7%)	71	5 (4.8%)	99	4 (3.8%)	100	2 (1.9%)	102	10 (9.6%)	94	0 (0%)	104	-	0	-	0	-	0	-	0	104
2007	70 (85.4%)	12	24 (29.3%)	58	19 (23.2%)	63	17 (20.7%)	65	5 (6.1%)	77	5 (6.1%)	77	1 (1.2%)	81	-	0	-	0	-	0	-	0	82
2008	42 (63.6%)	24	17 (25.8%)	49	5 (7.6%)	61	4 (6.1%)	62	1 (1.5%)	64	6 (9.1%)	59	0 (0%)	66	37 (56.1%)	29	21 (31.8%)	45	17 (25.8%)	49	0 (0%)	66	66^e^
2009	177 (83.9%)	34	45 (21.3%)	166	21 (10%)	190	17 (8.1%)	194	11 (5.2%)	200	11 (5.2%)	200	3 (1.4%)	208	133 (63.0%)	78	103 (48.8%)	108	55 (26.1%)	156	0 (0%)	211	211
2010	31 (77.5%)	9	6 (15.0%)	34	8 (20.0%)	32	6 (15.0%)	34	1 (2.5%)	39	2 (5.0%)	38	0 (0%)	40	25 (62.5%)	15	23 (57.5%)	17	6 (15.0%)	34	0 (0%)	40	40
2011	63 (76.8%)	19	14 (17.1%)	68	14 (17.1%)	68	11 (13.4%)	71	3 (3.7%)	79	3 (3.7%)	79	2 (2.4%)	80	48 (58.5%)	33	40 (48.8%)	42	15 (18.3%)	67	1 (1.2%)	81	82
2012	43 (89.6%)	5	7 (14.6%)	41	10 (20.8%)	38	8 (16.7%)	40	5 (10.4%)	43	1 (2.1%)	47	3 (6.2%)	45	26 (54.2%)	22	28 (58.3%)	20	13 (27.1%)	35	1 (2.1%)	47	48
2013	54 (78.3%)	15	8 (11.6%)	61	9 (13.0%)	60	4 (5.8%)	65	5 (7.2%)	64	1 (1.4%)	68	1 (1.4%)	68	40 (58.0%)	29	40 (58.0%)	29	19 (27.5%)	50	0 (0%)	69	69
2017	123 (75.0%)	41	35 (21.3%)	129	34 (20.7%)	130	21 (12.8%)	143	16 (9.8%)	148	-	0	2 (1.2%)	162	118 (72.0%)	46	110 (67.1%)	54	59 (36.0%)	105	1 (0.6%)	163	164
Total	1077 (81.7%)	241	331 (23.6%)	1007	165 (12.5%)	1153	124 (9.4%)	1194	91 (6.9%)	1226	61 (5.3%)	1092	23 (1.7%)	1295	427 (62.8%)	252	365 (53.7%)	315	184 (27.1%)	496	3 (0.4%)	667	1318

^a^In the dataset (D_am7) that included the antimicrobial susceptibility testing results for seven antimicrobials from 2001 to 2013

^b^In the dataset (D_am10) that contained the antimicrobial susceptibility testing results for 10 antimicrobials from 2008 to 2013 and 2017

^c^Resistant

^d^Susceptible

^e^From the data of 2008, one isolate had no information about the testing result of GEN, and one had no information about the testing result of FFC

*TET* tetracycline, *CHL* chloramphenicol, *CIP* ciprofloxacin, *NAL* nalidixic acid, *GEN* gentamicin, *FFC* florfenicol, *CTX* cefotaxime, *SMX* sulfamethoxazole, *AMP* ampicillin, *TMP* trimethoprim, *CAZ* ceftazidime

#### Descriptive analysis

Summary and descriptive analyses to determine the resistotypes and their relative frequencies were facilitated by the ‘tidyverse’ package [21]. This was also performed for the top four most common serotypes in pigs in the dataset, namely Typhimurium, 1,4,[5],12:i:-, Derby and Rissen, and Venn diagrams were built to explore the similarity between their resistotypes.

#### Spatial and temporal trends

The proportion of isolates with information on their geographical origin that was resistant to each of the 12 antimicrobials at the province level was calculated and later adjusted using empirical Bayesian smoothing with the ‘spdep’ package [22]. For the empirical Bayesian smoothing, the neighbouring relationships were described by Queen contiguity, considering two provinces as neighbours when they shared at least one point of their boundaries.

With the aim of jointly modelling the evolution over time of the proportion of resistant strains for each of the antimicrobials, generalized linear models were constructed, denoting by ${{\varvec{\pi}}}_{i}= {\pi }_{i1},\dots$, ${\pi }_{iJ}$, the probability for isolate $i$ to be resistant to antimicrobials 1…$J$. The considered model has the following functional form:

$$\text{logit(}{\pi }_{ij}\text{)}={{\varvec{x}}}_{{\varvec{i}}{\varvec{j}}}{\varvec{\beta}}$$

To account for the fact that AST results on the same isolate are not independent, the parameter estimates were not obtained through the classical maximum likelihood theory. Rather, the generalized estimating equations (GEE) approach was followed to incorporate the unknown correlation between the outcomes, facilitated by “gee” package [23]. The unstructured working correlation was chosen in order not to pose any restrictions on the underlying relations. An exhaustive search to obtain the best model describing the temporal trends for the antimicrobials was conducted for each dataset. The search considered the inclusion of an interaction between an antimicrobial and the linear year term and if the interaction held, the interaction with the quadratic year term. In total, 2,186 and 59,045 possible models were built for D_am7 and D_am10, respectively, and the model with the lowest Quasi Information Criterion (QIC) was selected as the best model for each dataset using “MuMIn” package [24, 25].

#### Bayesian network analysis

Bayesian networks were built for binary AST results using the R package ‘bnlearn’ [26]. Bayesian networks are graphical models where vertices represent random variables and arcs represent probabilistic dependencies between them [27]. Hill-Climbing greedy search was applied to identify directed network structure. Arcs were retained in the networks if their empirical frequency in 100,000 bootstrap samples was ≥ 40%. Four Bayesian networks were built for D_am7 with (a) all data, (b) data from 2001 to 2004, (c) data from 2005 to 2008, and (d) data from 2009 to 2013 to assess if the structure of the network changed over time. Similarly, for D_am10, three were constructed using (a) all information, (b) data from 2008 to 2010, and (c) data from 2011 to 2017, respectively.

#### Hierarchical clustering

Hierarchical clustering analysis was carried out for both binary logarithms of the MICs and binary AST results by using R packages ‘FactoMineR’, ‘factoextra’ and ‘missMDA’ [28–30]. The regularised iterative principal components analysis algorithm and the regularised iterative multiple correspondence analysis algorithm were used to impute missing AST results (2 isolates with one missing AST result each) and serotype information (16 isolates) in the numeric and binary data, respectively [30]. Hierarchical trees were constructed using Ward’s minimum variance method based on Euclidean distances [31], and the number of clusters was determined when the partition had the highest relative loss of inertia [32]. The composition of binary AST results of the antimicrobials and the serotype of the isolates were elicited for each cluster. The percentage of isolates of a cluster belonging to each specific province was depicted on the map, and the temporal trends in the percentage were also generated.

## Results

### Descriptive results

A total of 1,318 pig *Salmonella* isolates belonging to 63 different serotypes that were retrieved between 2001 and 2017 were included in at least one of the datasets (Table 1). The yearly number of isolates ranged from 40 to 211. D_am7 consisted of 1,154 isolates from 58 serotypes, of which 23.5% were Typhimurium, 21.2% were Rissen, 16.6% were Derby, 10.1% were 1,4,[5],12:i:-, 5.2% were Anatum, and 4.7% were Bredeney. Among the 1,154 isolates, 318 (27.6%) did not have geographical information. D_am10 included 680 isolates from 46 serotypes of which the most frequent were Rissen (23.9%), 1,4,[5],12:i:- (21.1%), Typhimurium (19.9%), Derby (14.1%), Anatum (2.5%) and Bredeney (2.5%). A total of 281 (41.3%) isolates had missing geographical information in D_am10. Trends in serotypes have been described elsewhere [16]. 

**Fig. 1:**
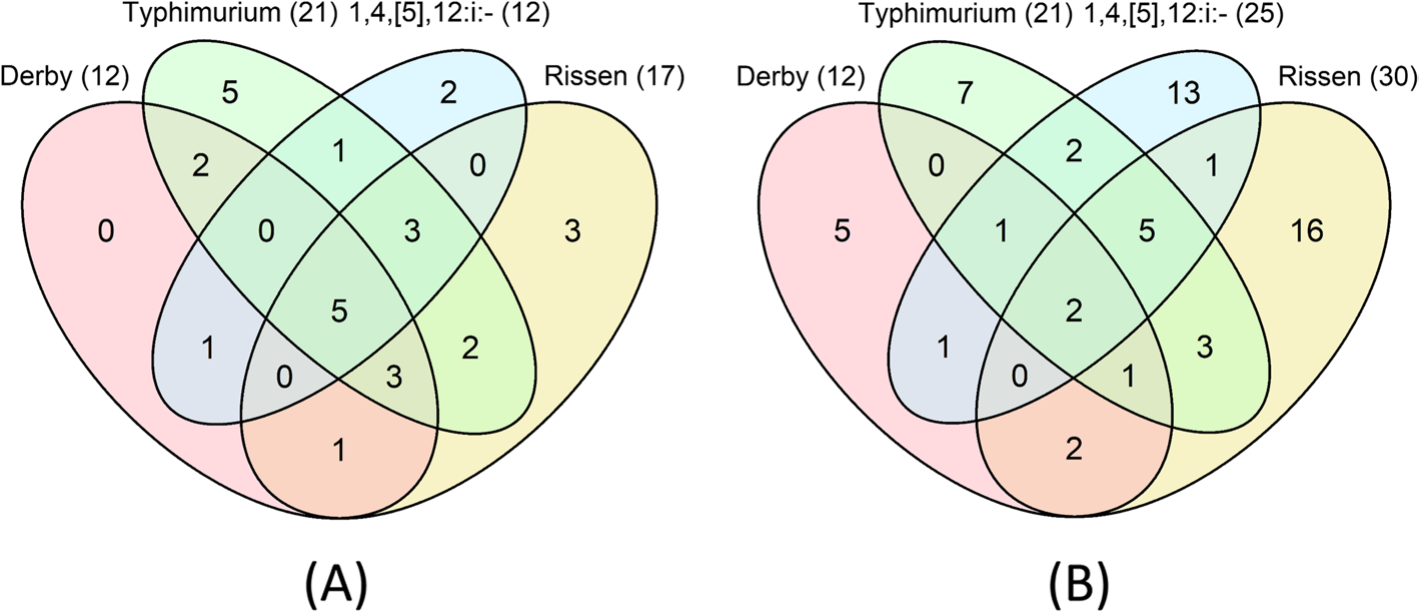
Venn diagrams illustrating the number of resistotypes in *Salmonella* isolates of serotypes Typhimurium, Rissen, Derby, and 1,4,[5],12:i:- from pigs recovered through the Spanish Veterinary Antimicrobial Resistance Surveillance Network programme. (**a**) contains 1,154 isolates with seven antimicrobial susceptibility results between 2001 and 2013 (D_am7); (**b**) contains 680 isolates with ten antimicrobial susceptibility results between 2008 and 2017 (D_am10)

Twenty-nine out of the 256 (2^7^) possible resistotypes in D_am7 were observed, (including 21 present in Typhimurium, 17 in Rissen, 12 in Derby, and 12 in 1,4,[5],12:i:- isolates) (Fig. 1). Almost 15% (n = 170) of the isolates were susceptible to all seven antimicrobials (Supplementary File 2). Isolates with a MDR resistotype (i.e., resistance to 3 or more antimicrobial classes) accounted for 8.9% (n = 103) of all isolates, and the annual percentage of MDR isolates decreased from 30.1% (n = 22) in 2001 to only 1.6 (n = 1) in 2008 (Table 2). However, it then increased to 7.2 − 10.4% in 2011 − 2013. Among the 607 (52.7%) isolates that were resistant to only one antimicrobial, 592 (51.4%) were resistant to TET, and 9 (0.8%) and 6 (0.5%) were resistant to CHL and CIP, respectively. In D_am10, 65 resistotypes were observed among the 1024 (2^10^) possible combinations of resistances (Supplementary File 2). There were 21 resistotypes in Typhimurium isolates, 30 in Rissen isolates, 15 in Derby isolates, and 25 in 1,4,[5],12:i:- isolates (Fig. 1). More than half of the resistotypes identified among Rissen and 1,4,[5],12:i:- isolates were specific to that serotype. Around 15% of the isolates (n = 103) were susceptible to all the antimicrobials, which is similar to the proportion in D_am7. However, there was a higher percentage of MDR isolates (54.0%, n = 366) due to the inclusion of two antimicrobials (SMX and AMP) against which high levels of resistance were found, and an increasing trend in the annual proportion of MDR isolates was observed (from 37.5% in 2008 to 62.8% in 2017; Table 2). Most isolates that were resistant to TET were also resistant to SMX and/or AMP, and just 14.2% (n = 96) were only resistant to TET.

**Table 2:** Percentage of multidrug-resistant *Salmonella* isolates (the number of multidrug-resistant *Salmonella* isolates/number of all *Salmonella* isolates) by year from pigs collected through the Spanish Veterinary Antimicrobial Resistance Surveillance Network programme that were tested for susceptibility to eleven antimicrobials from 2001 to 2017

**Year**	**D_am7** ^a^	**D_am10** ^b^
2001	30.1% (22/73)	
2002	15.6% (7/45)	
2003	10.7% (9/84)	
2004	6.3% (7/111)	
2005	8.6% (11/128)	
2006	3.8% (4/104)	
2007	12.8% (10/78)	
2008	1.6% (1/63)	37.5% (24/64)
2009	4.7% (10/211)	52.6% (111/211)
2010	5.0% (2/40)	57.5% (23/40)
2011	8.5% (7/82)	48.1% (39/81)
2012	10.4% (5/48)	56.2% (27/48)
2013	7.2% (5/69)	55.1% (38/69)
2017		62.8% (103/164)
Total	8.9% (103/1152)	54.0% (366/678)

^a^D_am7 included the antimicrobial susceptibility testing results for seven antimicrobials (tetracycline, chloramphenicol, ciprofloxacin, nalidixic acid, gentamicin, florfenicol, and cefotaxime) from 2001 to 2013;

^b^D_am10 contained the antimicrobial susceptibility testing results for 10 antimicrobials (tetracycline, chloramphenicol, ciprofloxacin, nalidixic acid, gentamicin, cefotaxime, sulfamethoxazole, ampicillin, trimethoprim and ceftazidime) from 2008 to 2013 and 2017

### Spatial trends

**Fig. 2:**
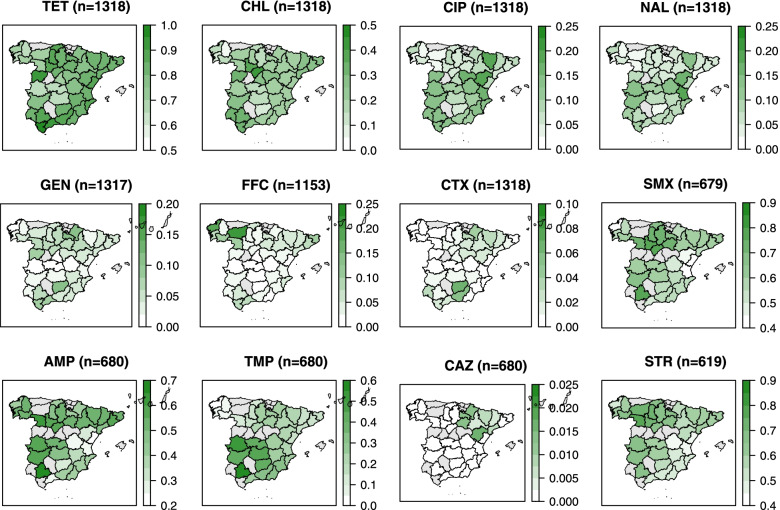
Spatial trends adjusted by empirical Bayesian smoothing in the proportion of *Salmonella* isolates from pigs resistant to twelve antimicrobials from 2001 to 2017, collected through the Spanish Veterinary Antimicrobial Resistance Surveillance Network programme. Provinces in grey indicate where there was no isolate *TET* tetracycline, *CHL* chloramphenicol, *CIP* ciprofloxacin, *NAL* nalidixic acid, *GEN* gentamicin, *FFC* florfenicol, *CTX* cefotaxime, *SMX* sulfamethoxazole, *AMP* ampicillin, *TMP* trimethoprim, *CAZ* ceftazidime, *STR* streptomycin

The empirically adjusted spatial distribution of the proportion of isolates resistant to each of the 12 antimicrobials is shown in Fig. 2 and Supplementary File 3. The percentage of isolates resistant to TET across Spain was the highest among all antimicrobials and ranged from 66.7% to 95.8%, followed by SMX (range: 42.5% − 77.8%), STR (range: 45.7% − 76.7%), AMP (range: 24.3% − 66.7% with a lower percentage of resistance in the South-East of Spain), and CHL (range: 8.5% − 41.1%). A higher proportion of isolates were resistant to TMP (range: 7.1% − 58.3%) in the southwest of Spain. The percentage of resistant isolates for CIP (range: 4.3% − 19.3%) and NAL (range: 3.1% − 18%) was lower in the northwest corner of Spain; in contrast, the value for FFC (range: 0% − 20.0%) was higher there.

#### Temporal trends

Detailed model results and work correlations can be found in Supplementary File 4.

#### D_am7

**Fig. 3:**
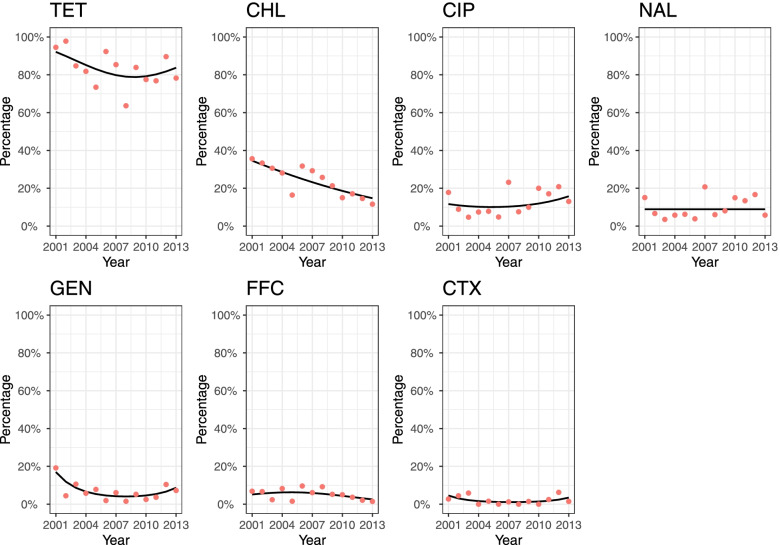
Temporal trends in the percentage of resistant *Salmonella* isolates from pigs collected through the Spanish Veterinary Antimicrobial Resistance Surveillance Network programme towards seven antimicrobials from 2001 to 2013. Red dots are the observed percentage of resistant isolates; black lines are the fitted values of generalized estimating equation models using the binary results (i.e., resistant and susceptible) *TET* tetracycline, *CHL* chloramphenicol, *CIP* ciprofloxacin, *NAL* nalidixic acid, *GEN* gentamicin, *FFC* florfenicol, *CTX* cefotaxime

A significant tempeoral change in the percentage of resistant isolates during 2001–2013 was found for all the antimicrobials except NAL. While the change for CHL was linear, a quadratic year term was included in the final models for TET, CIP, GEN, FFC and CTX (Fig. 3). According to the GEE estimates, a much higher percentage of isolates resistant to TET was expected (lowest in 2009 at 78.8% and highest in 2001 at 92.1%) than to other antimicrobials (median values <23.9%). From 2001 to 2013, the percentage of resistant isolates for TET, CIP and GEN first decreased and then increased again, while the odds of being resistant to CHL decreased by 8.9% [95% confidence interval (CI): 5.1 − 12.5%] each year. The predicted percentage of CTX and FFC resistant isolates remained below 10% for the most part.

#### D_am10

**Fig.4:**
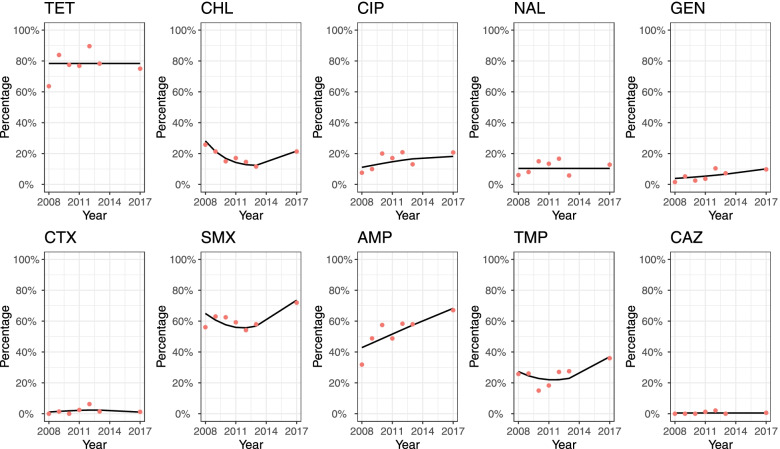
Temporal trends in the percentage of resistant *Salmonella* isolates from pigs collected through the Spanish Veterinary Antimicrobial Resistance Surveillance Network programme towards ten antimicrobials from 2008 to 2017. Red dots are the observed percentage of resistant isolates; black lines are the fitted values of generalized estimating equation models using the binary results (i.e., resistant and susceptible) *TET* tetracycline, *CHL* chloramphenicol, *CIP* ciprofloxacin, *NAL* nalidixic acid, *GEN* gentamicin, *CTX* cefotaxime, *SMX* sulfamethoxazole, *AMP* ampicillin, *TMP* trimethoprim, *CAZ* ceftazidime

A relatively high level of resistance to TET, SMX, AMP (median values >53.7%) compared to other antimicrobials (with median values <27.1%) was shown in the GEE predicted results. The GEE model found temporal changes in the percentage of resistant isolates between 2008 to 2017 for all the antimicrobials except TET, NAL and CAZ (Fig. 4), but the variation in the observed percentage of resistant isolates for TET was rather substantial. The percentage of isolates resistant to CIP, GEN, AMP increased over this period, and the odds of isolates being resistant to AMP was 1.12 times (95% CI: 1.08 − 1.17) higher than the previous year. Although the percentage of resistant isolates to CHL, SMX and TMP seemed to decrease from 2008 to 2013, the values in 2017 increased significantly. These observed and predicted figures for CTX and CAZ have remained below 10% over the years.

**Fig. 5:**
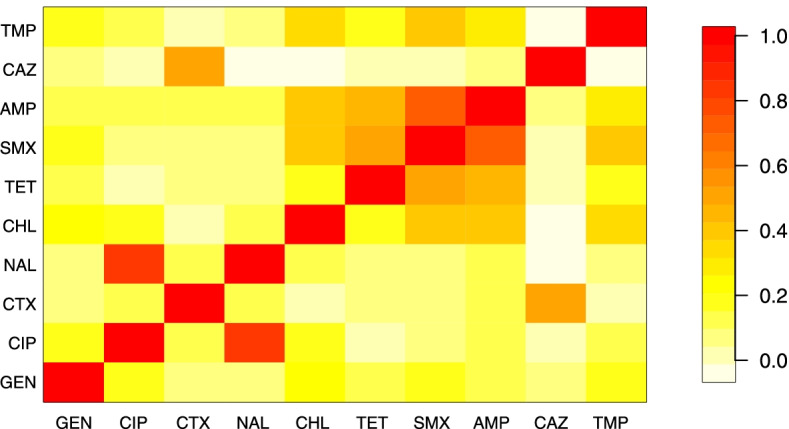
Estimated pairwise correlations obtained from the generalized estimating equations for the binary results (i.e., resistant and susceptible) of antimicrobial susceptibility testing for ten antimicrobials *TET* tetracycline, *CHL* chloramphenicol, *CIP* ciprofloxacin, *NAL* nalidixic acid, *GEN* gentamicin, *CTX* cefotaxime, *SMX* sulfamethoxazole, *AMP* ampicillin, *TMP* trimethoprim, *CAZ* ceftazidime

The estimates of the pairwise correlations obtained from the GEE modelling using D_am10 are shown in Fig. 5. As expected, the correlation coefficient for CIP and NAL was the highest among all pairs (0.82), and the correlation between CTX and CAZ was also relatively high (0.49). Among antimicrobials from different classes, SMX and AMP had a high correlation coefficient of 0.71, and both had relatively high correlation coefficients with TET, CHL and TMP (range: 0.33–0.51). GEN did not appear to be correlated with any other antimicrobials.

### Bayesian network analysis

**Fig. 6:**
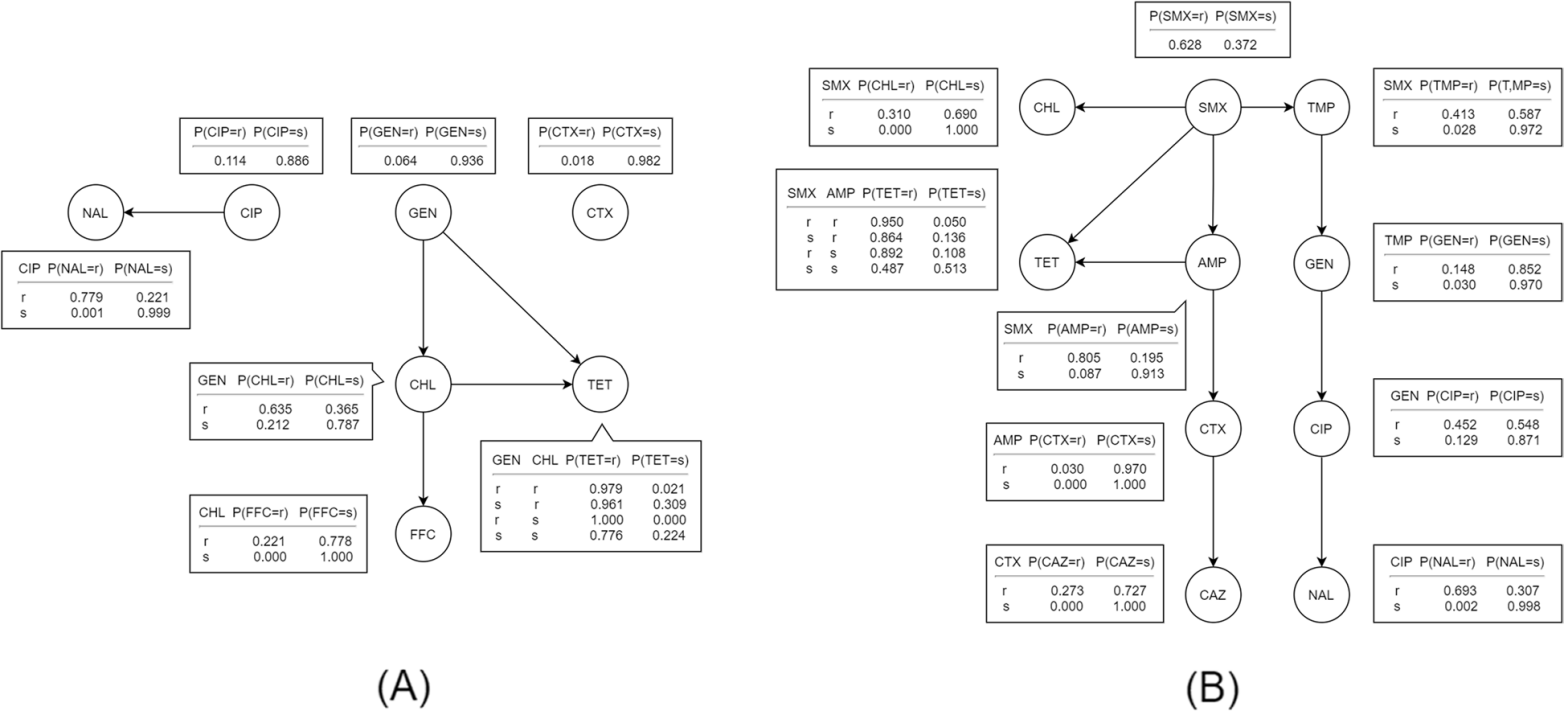
Bayesian networks for (**A**) seven and (**B**) ten binary antimicrobial susceptibility testing results of *Salmonella* isolates from pigs collected through the Spanish Veterinary Antimicrobial Resistance Surveillance Network programme between 2001 and 2013 for (**A**) and between 2008 and 2017 for (**B**). TET: tetracycline; CHL: chloramphenicol; CIP: ciprofloxacin; NAL: nalidixic acid; GEN: gentamicin; FFC: florfenicol; CTX: cefotaxime; SMX: sulfamethoxazole; AMP: ampicillin; TMP: trimethoprim; CAZ: ceftazidime; r: resistant; s: susceptible

The overall Bayesian networks identified for D_am7 and D_am10 are presented here (Fig. 6), and networks using data from specific periods for both D_am7 and D_am10 are shown in Supplementary File 5. The strength and the direction of the arcs in each of the networks can be found in Supplementary File 6.

For D_am7, five relations were built with CHL located in a central position. Except the relation between GEN-TET, all the others were also maintained in at least one of the partial networks (Supplementary File 5). A strong relationship between resistance to certain pairs of antimicrobials was identified in the overall network. The conditional probability for an isolate susceptible to CIP that was also susceptible to NAL was 0.999 but for an isolate resistant to CIP that was also resistant to NAL was only 0.779. An isolate susceptible to FFC would be expected to be susceptible to CHL, whereas an isolate resistant to CHL had a conditional probability of being resistant to FFC at only 0.221.

Regarding D_am10, SMX was located at the centre of the overall Bayesian network with 10 edges. Associations between CIP and NAL and between SMX and TET, CHL, AMP and TMP also persisted in all other partial networks. According to the overall D_am10 Bayesian network, the conditional probability for an isolate that was susceptible to SMX also being susceptible to CHL was 1 and to TMP was 0.972, while one that was resistant to SMX had a 0.310 probability of being resistant to CHL and a 0.413 probability of being resistant to TMP. An isolate resistant to CAZ would be expected to be resistant to CTX. Although networks of D_am7 suggested the occurrence of associations between resistance to CHL and resistance to TET and GEN, in the D_am10-based networks (which included more antimicrobials but fewer isolates), resistance to CHL became independent from TET and more dependent on the patterns for SMX and TMP which were not included in D_am7.

#### Hierarchical clustering

For D_am7, three and six clusters were generated using binary logarithms of the MICs and binary AST results, respectively, while the number of clusters identified for D_am10 was three and four. Here we present the hierarchical clustering results using binary AST information, and results of the analysis using binary logarithms of the MICs are shown in Supplementary File 7).

### D_am7

**Fig. 7:**
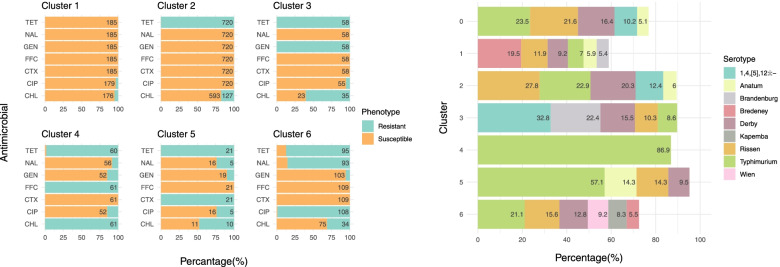
Hierarchical clusters using the binary antimicrobial susceptibility testing results of seven antimicrobials among 1,154 *Salmonella* isolates from pigs collected through the Spanish Veterinary Antimicrobial Resistance Surveillance Network programme between 2001 and 2013 (left: the proportion of isolates resistant to seven antimicrobials; right: the composition of serotypes in each of the clusters). Cluster 0 shows the serotype distribution of all isolates in the dataset (D_am7). Only serotypes accounting for ≥5% of the isolates in each particular cluster are shown in the graph

Six clusters with 185 (16.0%), 720 (62.4%), 58 (5.0%), 61 (5.3%), 21 (1.8%) and 109 (9.4%) isolates, respectively, were identified using the binary AST results in D_am7 (Fig. 7). Isolates in Cluster (n = 185, 16.0% of isolates in D_am7) were susceptible to almost all seven antimicrobials (with a few resistant to either CHL and/or CIP) (Fig. 7). The most abundant serotype in this cluster was Bredeney (36 isolates, accounting for two-thirds of all Bredeney isolates in D_am7), and several other serotypes including Montevideo, Enteritidis or Infantis were also overrepresented (i.e., with a percentage higher than the expected value in the cluster). In contrast, the most prevalent serotypes in the D_am7 collection, including Rissen, Derby, Typhimurium and especially 1,4,[5],12:i:- were under-presented in Cluster 1 (with only between 2 and 22 isolates from each). Cluster 2 was the largest (n = 720, 62.4%) and was characterized by resistance to TET and susceptibility to all other antimicrobials except CHL (17.6% resistance, n = 127). Serotype distribution in Cluster 2 was similar to that observed in the whole D_am7 panel with Rissen (27.8%; n = 200) and Derby (20.3%; n = 146) being overrepresented and Bredeney being underrepresented (1.5%, n = 11).

Resistance to both TET and GEN was the predominant feature present in all isolates in Cluster 3 (n = 58, 5.0%), with close to 60% of them being also resistant to CHL (n = 35). The most common serotypes in this cluster were 1,4,[5],12:i:- (32.8%; n = 19) and Brandenburg (22.4%; n = 13, representing 41.9% of all Brandenburg isolates in D_am7). Close to all isolates in Cluster 4 (n = 61, 5.3%) were resistant to TET, CHL and FFC and susceptible to CTX, and 85 − 92% of isolates (n = 52 − 56) were susceptible to CIP, NAL and GEN. This cluster was dominated by Typhimurium (86.9%; n = 53) isolates. Isolates in Cluster 5 (n = 21, 1.8%) were resistant to TET and CTX and susceptible to FFC, with varying resistance levels to other antimicrobials. Typhimurium accounted for 57.1% (n = 12) of the isolates in the cluster; 14.3% (n = 3) were Anatum and Rissen, respectively, and 9.5% were Derby (n = 2). Cluster 6 consisted of 109 isolates (9.4% of D_am7), including isolates that were typically resistant to CIP, NAL and TET and susceptible to GEN, FFC and CTX. Wien (9.2%; n = 10), Kapemba (8.3%; n = 9), Brikama (4.6%; n = 5) were overrepresented in Cluster 6, and the only isolates of Essen (n = 3) and Choleraesuis (n = 2) in D_am7 were in this cluster.

**Fig. 8:**
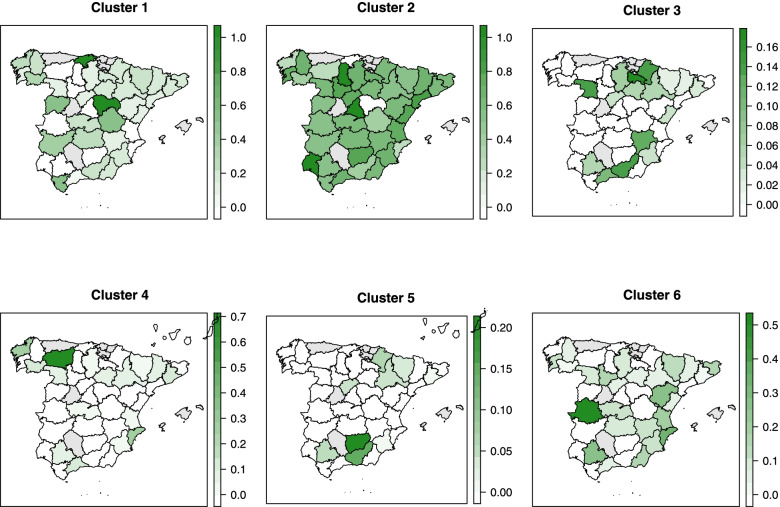
Spatial distribution of six clusters elicited from hierarchical clustering using binary antimicrobial susceptibility testing results of 836 *Salmonella* isolates from pigs collected through the Spanish Veterinary Antimicrobial Resistance Surveillance Network programme between 2001 and 2013. Provinces in grey indicate where there was no isolate

**Fig. 9:**
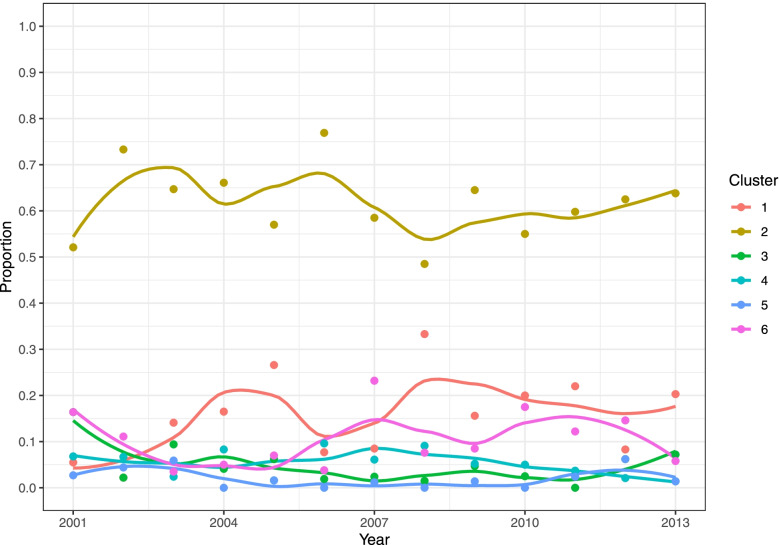
Temporal distribution of six clusters elicited from hierarchical clustering using binary antimicrobial susceptibility testing results of 836 *Salmonella* isolates from pigs collected through the Spanish Veterinary Antimicrobial Resistance Surveillance Network programme between 2001 and 2013

The spatial distribution of the proportion of isolates belonging to each of the clusters is shown in Fig. 8. While no obvious spatial patterns were observed for Clusters 1 and 2, a higher proportion of isolates of Cluster 3 was seen in the North and South of Spain. Northern provinces and provinces adjacent to the Mediterranean Sea had a larger proportion of isolates of Cluster 4. Some provinces in the Northeast and the South harbored more isolates belonging to Cluster 5. Last, the proportion of isolates of Cluster 6 seemed lower in the West of Spain, except the province Cáceres. No clear temporal trends in the proportion of isolates in different clusters were noted (Fig. 9).

### D_am10

**Fig. 10:**
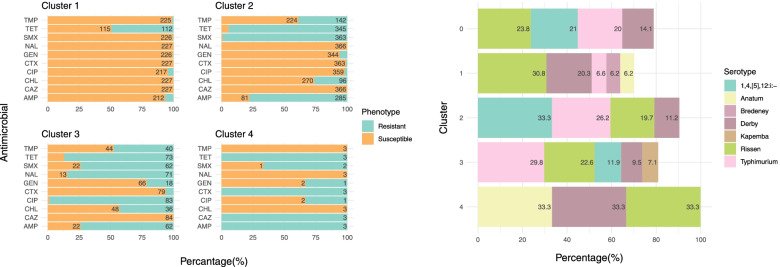
Hierarchical clusters using the binary antimicrobial susceptibility testing results of ten antimicrobials among 680 *Salmonella* isolates from pigs collected through the Spanish Veterinary Antimicrobial Resistance Surveillance Network programme between 2011 and 2017 (left: the proportion of isolates resistant to ten antimicrobials; right: the composition of serotypes in each of the clusters). Cluster 0 shows the serotype distribution of all isolates in the dataset (D_am10). Only serotypes accounting for ≥5% of the isolates in each particular cluster are shown in the graph

Four clusters with 227 (33.4%), 366 (53.8%), 84 (12.4%) and 3 (0.4%) isolates, respectively, were identified using the binary AST results in D_am10. In Cluster 1, half of the isolates were resistant to TET, and most were susceptible to the rest of the antimicrobials. Compared to the percentage of isolates of different serotypes in the whole D_am10, Cluster 1 had higher percentage of Rissen (30.8%; n = 70) and Derby (20.3%; n = 46) and much lower percentage of Typhimurium (6.6%; n = 15) and 1,4,[5],12:i:- (4.8%; n = 11) (Fig. 10). All the Montevideo (n = 7) and Infantis (n = 5) isolates and 3 out of the 5 Enteritidis isolates in D_am10 belonged to this cluster. Most of the isolates in Cluster 2 were resistant to AMP, SMX and TET, and susceptible to CIP, NAL, GEN, CTX, and CAZ. This cluster was dominated by 1,4,[5],12:i:- (n = 122; 33.3%) and Typhimurium (n = 96; 26.2%), and more than 90% of the Wien isolates (n = 10) were in this cluster. Cluster 3 was different from Cluster 2 in the addition of very high (> 84.5%) levels of resistance to CIP and NAL. The percentage of Rissen, Derby and Typhimurium was similar to the one of Cluster 2, but 1,4,[5],12:i:- only accounted for 11.9% (n = 10) of the isolates. All the Kapemba isolates (n = 6) in D_am10 belonged to this cluster. Finally, Cluster 4, which included only three isolates (all from different serotypes), was mainly characterized by full resistance to CAZ.

**Fig. 11:**
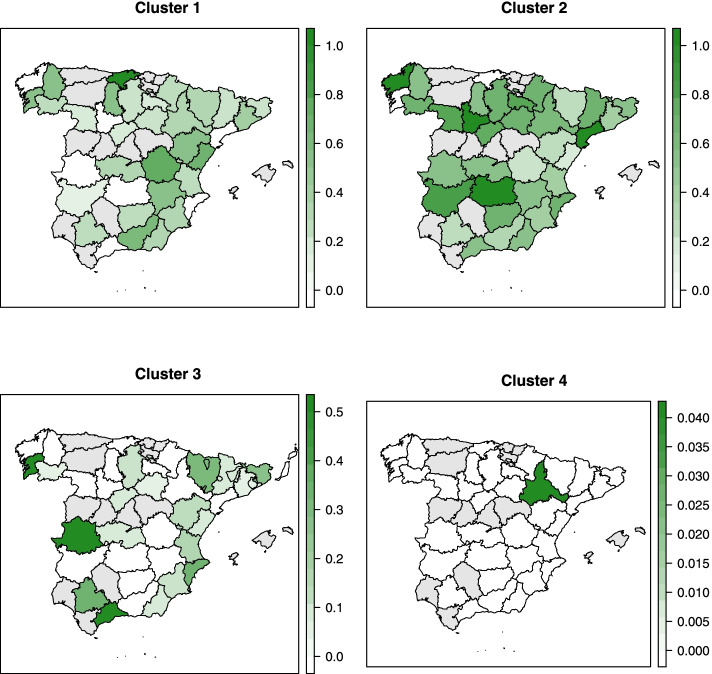
Spatial distribution of six clusters elicited from hierarchical clustering using binary antimicrobial susceptibility testing results of 399 *Salmonella* isolates from pigs collected through the Spanish Veterinary Antimicrobial Resistance Surveillance Network programme between 2011 and 2017. Provinces in grey indicate where there was no isolate

**Fig. 12:**
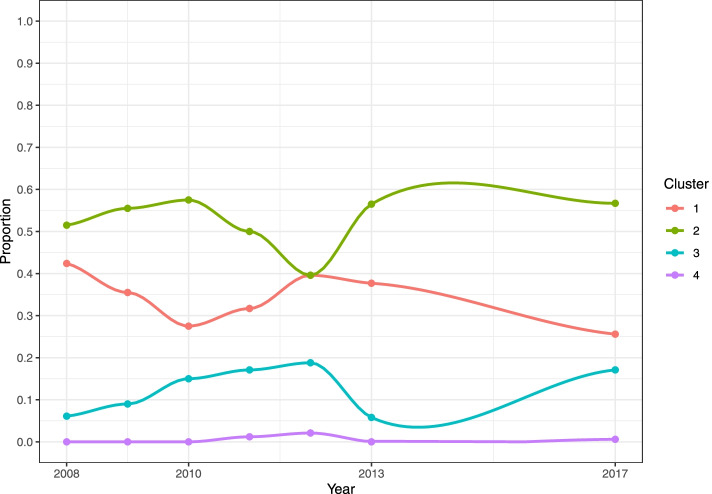
Temporal distribution of six clusters elicited from hierarchical clustering using binary antimicrobial susceptibility testing results of 399 *Salmonella* isolates from pigs collected through the Spanish Veterinary Antimicrobial Resistance Surveillance Network programme between 2011 and 2017

Figure 11 shows the proportion of isolates originating from different provinces in Spain in different clusters. Although there were no isolates from many of the provinces in the West, some patterns might be observed. A higher proportion of isolates in Cluster 1 was in the East than in the West, which seemed to be the opposite for Cluster 2. The proportion of isolates in Cluster 3 was higher in the far East and the far West of Spain, and all the isolates in Cluster 4 were in a single province of the west. The temporal trends in the proportion of isolates in the clusters seemed to oscillate over the period (Fig. 12).

## Discussion

The current study demonstrates the usefulness of a range of methods that can be easily applied to transform phenotypical AST data into information helpful in assessing the evolution of AMR over time, including temporal and spatial trends, and pairwise and multiple associations [33]. Some studies have previously presented multidrug-resistant patterns for* Salmonella* isolates recovered from pigs in (part of) Spain and elsewhere [9, 11, 34–37]. Although some AMR studies using multi-sourced *Salmonella* isolates have applied some multivariate analyses to explore the relations between the isolates [38, 39], to our best knowledge, reports of longitudinal AMR data from swine *Salmonella* isolates in Spain using multivariate analyses to explore links between resistances to specific antimicrobials were missing.

Two multivariate analyses, Bayesian network analysis and hierarchical clustering were conducted to investigate potential relationships between and patterns among the resistances to antimicrobials, whose existence was also demonstrated by the pairwise correlations from the GEE. Bayesian network analysis provides conditional probabilities of resistance to an antimicrobial given one or more resistances to other antimicrobials. This allows to uncover co-resistance patterns among the antimicrobials of interest efficiently and to discern the degree to which the resistance of an antimicrobial is associated with another one. However, although directionality was indicated in our Bayesian networks, causality should not be implied. Changes in Bayesian network structures and conditional probabilities between or among antimicrobials may indicate shifts in mechanisms of co-resistance among antimicrobials over time or space. Some of these changes were observed in the networks built with subsets of data of different periods while certain patterns were more stable. In any case, the volume of our data might have been insufficient to exhibit meaningful temporal trends. On the other hand, hierarchical clustering provides a rather straightforward way to explore common resistotypes whose spatial and temporal trends and dominant serotypes can be subsequently inspected. When this method was applied to both the binary logarithm of MIC and binary AST results, the results showed that, although the optimal number of clusters found might be different, the composition of clusters was similar if the number of clusters was the same. For example, the composition of three clusters generated using both types of data of D_am10 was very alike. However, when the number of clusters increased to four for D_am10, a CAZ-resistant cluster and a GEN-AMP resistant cluster were found using the binary logarithm of MICs and the binary AST results, respectively.

In our data, a large proportion of the isolates (>53.7%) were resistant to TET, SMX or AMP, fewer isolates (9.4 − 27.1%) were resistant to CHL, CIP, NAL or TMP, and very few isolates (<6.9%) were resistant to GEN, FFC, CTX, or CAZ. The resistance profile of *Salmonella* revealed by the current study was notably different from the ones in other countries in Europe [12], the United States [40] and China [41], although TET resistance seemed to be the most prominent in all the countries. There was a 4-year gap between 2013 and 2017, and the percentage of resistant isolates to some antimicrobials such as SMX, AMP and TMP increased substantially during this period. It is not clear whether this increase reflects the true situation in the field as the results of 2015 and 2019 were unavailable to us (The Spanish Veterinary Antimicrobial Resistance Surveillance Network programme started to survey AMR in *Salmonella* in pigs every odd year since 2013). When comparing with the AST results of the isolates from clinical human salmonellosis cases in Spain from 2010 to 2017, the resistance levels to all antimicrobials except FFC (not tested in human isolates), CTX, and CAZ were substantially higher [42–48]. Still, similar temporal trends in the percentage of resistant isolates from swine (this study) and humans [42–48] were observed for TET and CHL (decreasing trend) and CIP (increasing trend) but not for other antimicrobials, which may suggest the existence of other sources of foodborne salmonellosis for the general public in Spain in which antimicrobial resistance dynamics may be different.

The values of and trends in the percentage of isolates with MDR resistance varied substantially between D_am7 and D_am10, which could be explained by the inclusion of SMX and AMP, two antimicrobials in which high levels of resistance were observed in D_am10. Except in 2008, the proportion of MDR isolates in D_am10 was always between 50–60% (Table 2), while a clear decreasing trend in the percentage of clinical human MDR isolates (about 50% in 2012 to about 25% in 2017) has been reported in the same time frame [42–47].

The relationships revealed by Bayesian networks largely agreed with the clustering patterns found by hierarchical clustering and were expected in certain cases, such as the CIP and NAL, CHL and FFC, and CTX and CAZ links. Interestingly, although different antimicrobials were used in D_am7 and D_am10, similar patterns were still discovered by hierarchical clustering. These included a cluster of mostly pan-susceptible isolates (although about half of the isolates were resistant to TET in this D_am10 cluster), a cluster of isolates resistant to CIP and NAL (and TET) and a cluster of isolates with resistance to GEN (and TET and to a lesser extent CHL) (for D_am10, this pattern was shown in the results using binary AST information with a pre-set cluster number). Clusters of resistance to CHL and FFC (which was not in D_am10) and to CTX were also observed in D_am7, and a cluster with isolates resistant to CAZ (which was not in D_am7) was identified in D_am10. Antimicrobials that did not show clear patterns in the hierarchical clusters─ CHL in D_am7 and SMX, AMP, and TMP in D_am10─ were located in a more central position in the Bayesian networks.

The strong linkage between resistance to CIP and NAL (found in all analyses carried out) was independent of the resistance to other antimicrobials according to the Bayesian network analyses, and isolates resistant to CIP and NAL were classified in the same cluster in both D_am7 (Cluster 6) and D_am10 (Cluster 4). These results indicate that mechanisms of the resistance of CIP and NAL are independent of the resistance to other antimicrobials, which points out the existence of chromosomal mutations in the quinolone resistance determinant regions (not related to the horizontal transfer of resistance determinants along with resistance genes to other antimicrobial families). However, a small proportion (22.1%) of NAL-susceptible or CIP-resistant isolates were also found. This atypical quinolone resistance phenotype has been linked to the carriage of plasmid‐mediated quinolone resistance genes [49, 50]. The association in the presentation of fluoroquinolone resistance with resistance to aminoglycosides (as suggested in the Bayesian network for D_am10) and β-lactams and sulfonamides (as indicated in the clustering analysis) could suggest the involvement of mechanisms other than chromosomal mutations in at least a proportion of the strains, although this hypothesis should be confirmed using molecular tools such as whole-genome sequencing.

A strong correlation between the resistance phenotype of FFC and CHL, both belonging to the phenicol family, was also found as expected, and could be mediated by genes conferring resistance to both antimicrobials such as the floR gene. However, approximately 80% of the CHL-resistant genes were susceptible to FFC, suggesting the presence of other genes whose presence do not result in resistance to FFC, including cat and cmlA [10, 51–53].

In D_am10, resistotypes with a core pattern of TET, SMX, and AMP were most common in Typhimurium (83.0%) and its monophasic variant (78.3%) (and this could also be true for isolates in D_am7 for which SMX and AMP were not tested). This could indicate isolates carried the resistance background typical of DT104 as, among AMP-SMX-TET resistant isolates that were also tested for STR, 93.5% were also resistant to STR. The AMP-SMX-TET resistance pattern was also found in more than 40% of the Rissen isolates, in agreement with previous AMR results of *Salmonella* from pigs in Spain from 2003 to 2004 [9]. A similar AMP-CHL-SMX-TET-TMP pattern has been also described as very prevalent among Derby isolates from poultry [54], while the majority of the core resistant pattern of our Derby isolates was TET-SMX, thus suggesting the circulation of different strains/resistance genes in the different host species as previously suggested for Derby isolates circulating in pig and poultry populations in France [55].

The results of hierarchical clustering demonstrate the existence of serotype-specific differences in the presentation of several resistance patterns: For example, 1,4,[5],12:i:- isolates were more likely associated with MDR profiles, with no isolate presenting pan-susceptible profiles and most (92.3%) of the isolates in D_am10 being classified in highly resistant clusters (e.g., Cluster 2 and 3). In contrast, all Montevideo isolates were pan-susceptible, and most 65 – 100%) of the isolates belonging to serotypes Enteritidis, Infantis and Bredeney were classified in the “susceptible” clusters in D_am7 (Cluster 1) and D_am10 (Cluster 1). These results provide directions for further exploration of patterns of AMR within isolates of specific serotypes.

There are some limitations to the current study. First, due to the difference in the antimicrobials tested, we could not analyse the data as a whole nor every test result. Second, as the panels used for AST did not remain the same across the years, the range of inhibitory concentrations of some antimicrobials changed. This made the results of hierarchical clustering using the binary logarithm of MIC less reliable. Also, the graphical information of some *Salmonella* isolates was missing, compromising the reliability of the results of the spatial analysis. Furthermore, since samples were collected at abattoirs of high slaughter capacity (adding up to >50% of the national slaughter capacity), the results might not be representative of the farms that did not usually work with these abattoirs. Nevertheless, this strategy is in fact in line with the EU regulations regarding sampling strategies for monitoring and reporting of AMR in zoonotic and commensal bacteria, and the inclusion of a large number of samples throughout the study period and the consistency in the tests performed for at least several periods allowed the detection of certain robust trends in our collection.

## Conclusion

Our study demonstrated the power of multivariate statistical methods such as Bayesian network analysis and hierarchical clustering in combination with spatial and temporal analyses applied to phenotypical AMR data for generating valuable insights about patterns of and associations between AMR phenotypes. Besides spatial and temporal trends in the percentage of isolates being resistant to various antimicrobials, we also found pairwise relationships between antimicrobials and patterns of resistotypes. The existence of serotype-specific AMR patterns for serotypes of public health concern in *Salmonella* isolates in pigs in Spain allows us to draw hypotheses on their possible genetic background. Future research should focus on testing these hypotheses using highly discriminatory molecular tools to decipher the relationship between strains and resistance patterns found in *Salmonella* from different origins.

## Supplementary Information

**Additional file 1.**

**Additional file 2.**

**Additional file 3.**

**Additional file 4.**

**Additional file 5.**

**Additional file 6.**

**Additonal file 7.**

**Additional file 8.**

## Data Availability

The individual-level data is provided as Supplementary File 8.
