# Supplementary material for: Patterns of antimicrobial resistance in Salmonella isolates from fattening pigs in Spain
Source: BMC Vet Res. 2022 Sep 3;18:333. doi: 10.1186/s12917-022-03377-3 (PMC9440507; doi:10.1186/s12917-022-03377-3)
Supplement: Supplementary file 1 — Additional file 1. [file 12917_2022_3377_MOESM1_ESM.docx]

**Supplementary File 1.** The EUCAST epidemiological cut-off value (ECOFFs) and the ranges for each antimicrobial and year.

| **Antimicrobial** | **ECOFF** | **2001–2007** | **2008–2013** | **2017** |
| --- | --- | --- | --- | --- |
| Tetracycline | 8 | 0.5–256 | 1–64 | 2–64 |
| Chloramphenicol | 16 | 2–256 | 2–64 | 8–128 |
| Ciprofloxacin | 0.064 | 0.06–32 | 0.008–8 | 0.016–8 |
| Nalidixic acid | 16 | 0.5–128 | 4–64 | 4–128 |
| Gentamicin | 2 | 0.25–64 | 0.25–32 | 0.5–32 |
| Florfenicol | 16 | 1–32 (2001)  2–64 (2002–2006)  4–128 (2006–2007) | 2–64 | – |
| Cefotaxime | 0.5 | 0.03–4 | 0.06–4 | 0.25–4 |
| Sulfamethoxazole | 256 | – | 8–1024 | |
| Ampicillin | 8 | – | 0.06–32 | 1–64 |
| Trimethoprim | 2 | – | 0.5–32 | 0.25–32 |
| Ceftazidime | 2 | – | 0.25–16 | 0.25–128 |
