# Supplementary material for: Patterns of antimicrobial resistance in Salmonella isolates from fattening pigs in Spain
Source: BMC Vet Res. 2022 Sep 3;18:333. doi: 10.1186/s12917-022-03377-3 (PMC9440507; doi:10.1186/s12917-022-03377-3)
Supplement: Supplementary file 5 — Additional file 5. [file 12917_2022_3377_MOESM5_ESM.docx]

**Supplementary File 5**. Bayesian networks analysis results.


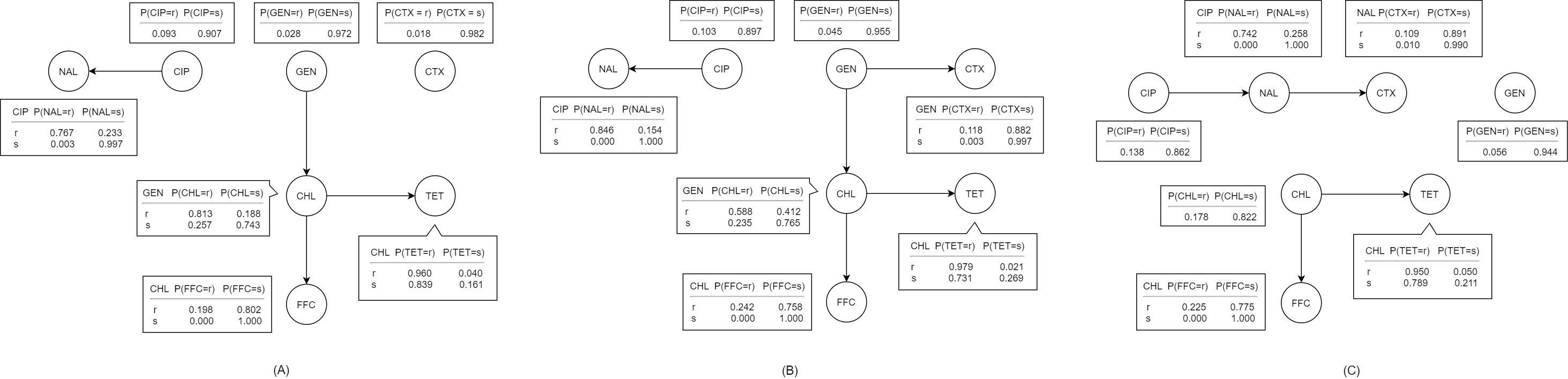


**Supplementary Figure 5.1.** Bayesian networks for seven binary antimicrobial susceptibility testing results of *Salmonella* isolates from pigs collected through the Spanish Veterinary Antimicrobial Resistance Surveillance Network programme between 2001 and 2013. (A) used data from 2001 to 2004, (B) used data from 2005 to 2008, and (C) used data from 2009 to 2013. CHL: chloramphenicol; CIP: ciprofloxacin; CTX: cefotaxime; FFC: florfenicol; GEN: gentamicin; NAL: nalidixic acid; TET: tetracycline; r: resistant; s: susceptible.


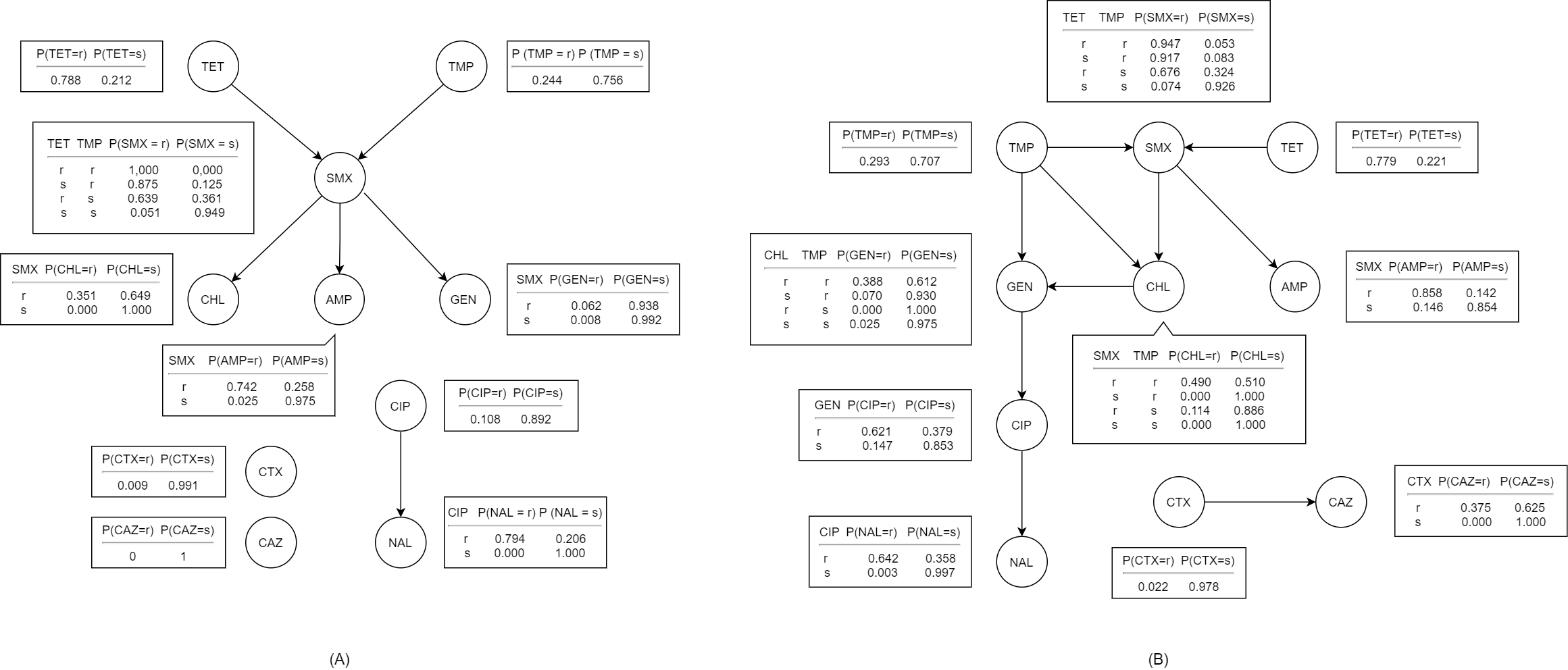


**Supplementary Figure 5.2.** Bayesian networks for ten binary antimicrobial susceptibility testing results of *Salmonella* isolates from pigs collected through the Spanish Veterinary Antimicrobial Resistance Surveillance Network programme between 2008 and 2017. (A) used data from 2008 to 2010, and (B) used data from 2011 to 2017. AMP: ampicillin; CAZ: ceftazidime; CHL: chloramphenicol; CIP: ciprofloxacin; CTX: cefotaxime; GEN: gentamicin; NAL: nalidixic acid; SMX: sulfamethoxazole; TET: tetracycline; TMP: trimethoprim; r: resistant; s: susceptible
