# Supplementary material for: Patterns of antimicrobial resistance in Salmonella isolates from fattening pigs in Spain
Source: BMC Vet Res. 2022 Sep 3;18:333. doi: 10.1186/s12917-022-03377-3 (PMC9440507; doi:10.1186/s12917-022-03377-3)
Supplement: Supplementary file 7 — Additonal file 7. [file 12917_2022_3377_MOESM7_ESM.docx]

**Supplementary File 7.** Hierarchical cluster results using binary logarithms of the minimum inhibitory concentrations (MICs) for both two datasets D_am7 and D_am10. D_am7 included the antimicrobial susceptibility testing results for seven antimicrobials (TET: tetracycline, CHL: chloramphenicol, CIP: ciprofloxacin, NAL: nalidixic acid, GEN: gentamicin, FFC: florfenicol and CTX: cefotaxime) from 2001 to 2013. D_am10 contained the antimicrobial susceptibility testing results for 10 antimicrobials (TET, CHL, CIP, NAL, GEN, CTX, SMX: sulfamethoxazole, AMP: ampicillin, TMP: trimethoprim and CAZ: ceftazidime) from 2008 to 2013 and 2017.


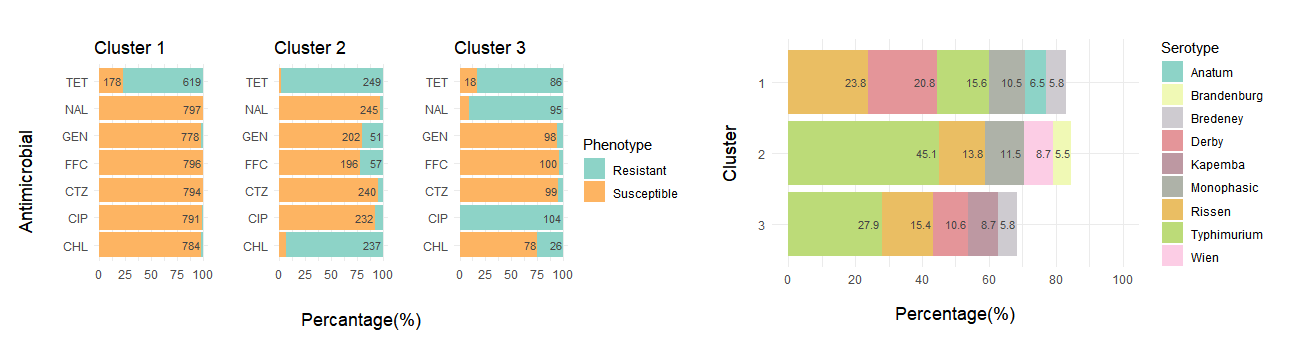
Three clusters with 797 (69.1%), 253 (21.9%) and 104 (9.0) isolates, respectively, were detected using binary logarithms of the MICs for D_am7 (Supplementary Fig. 7.1). In Cluster 1, 77.4% of the isolates were resistant to TET, and 95.0% were susceptible to all the remaining six antimicrobials. Cluster 2 was characterized by a high percentage of isolates resistant to both TET and CHL (92.1%), and Cluster 3 included isolates that were typically resistant to TET, CIP and NAL (77.9% resistant to all three antimicrobials). Rissen, Derby, Typhimurium and 1,4,[5],12:i:- serotypes accounted for 23.8%, 20.8%, 15.6% and 10.5% of the isolates in Cluster 1, respectively, and they were also the dominant serotypes in Cluster 3, apart from 1,4,[5],12:i:-. Typhimurium (45.1%) and its monophasic variant (11.5%) composed a great percentage of the isolates in Cluster 2.

**Supplementary Figure 7.1**. Hierarchical clusters using the binary logarithm of minimum inhibitory concentration results of seven antimicrobials of 1,154 *Salmonella* isolates from pigs collected through the Spanish Veterinary Antimicrobial Resistance Surveillance Network programme between 2001 and 2013 (left: the proportion of isolates resistant to seven antimicrobials; right: the composition of serotypes in each of the clusters). Only serotypes accounting for ≥ 5% of the isolates in each particular cluster are shown in the graph

Using the binary logarithm of MICs of 10 antimicrobials in D_am10, three clusters with 227 (33.4%), 358 (52.6%) and 95 (14.0%) isolates, respectively, were identified (Supplementary Fig. 7.2). In Cluster 1, half of the isolates were resistant to TET, and all were susceptible to the rest of the antimicrobials. In terms of serotypes, 29.5% (n=67) and 21.6% (n=49) of the isolates were Rissen and Derby, respectively, and Typhimurium and 1,4,[5],12:i:- only accounted for 6.6% (n=15) and 3.1% (n=7). All the Montevideo (n=7), Enteritidis (n=6) and Infantis (n=5) isolates and more than 80% of the Bredeney and Anatum isolates in D_am10 belonged to this cluster. Most of the isolates in Cluster 2 were resistant to TET, SMX and AMP and susceptible to CIP, NAL, CTX, and CAZ. This cluster was dominant by 1,4,[5],12:i:- (n=121; 33.8%) and Typhimurium (n=94; 26.3%), and more than 90% of the Wien isolates (n=10) in D_am10 were in this cluster. The main difference between Cluster 3 and 2 was that most isolates were resistant to CIP and NAL in Cluster 3. The composition of the serotypes in Cluster 3 was similar to the one of D_am10, but all the Kapemba isolates in D_am10 belonged to this cluster.


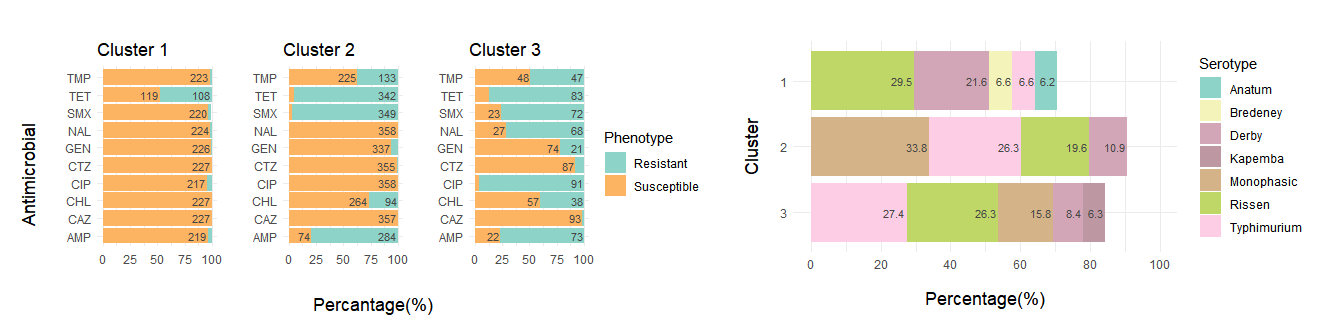


**Supplementary Figure 7.2.** Hierarchical clusters using the binary logarithm of minimum inhibitory concentration results of ten antimicrobials of 680 *Salmonella* isolates from pigs collected through the Spanish Veterinary Antimicrobial Resistance Surveillance Network programme between 2008 and 2017 (left: the proportion of isolates resistant to ten antimicrobials; right: the composition of serotypes in each of the clusters). Only serotypes accounting for ≥ 5% of the isolates in each particular cluster are shown in the graph
